# Structural Features of Patients with Drusen-like Deposits and Systemic Lupus Erythematosus

**DOI:** 10.3390/jcm11206012

**Published:** 2022-10-12

**Authors:** Marc Kukan, Matthew Driban, Kiran K. Vupparaboina, Swen Schwarz, Alice M. Kitay, Mohammed A. Rasheed, Catharina Busch, Daniel Barthelmes, Jay Chhablani, Mayss Al-Sheikh

**Affiliations:** 1 Department of Ophthalmology, University Hospital Zurich, University of Zurich, Frauenklinikstrasse 24, 8091 Zurich, Switzerland; 2 UPMC Eye Center, University of Pittsburgh, Pittsburgh, PA 15213, USA; 3 School of Optometry and Vision Science, University of Waterloo, Waterloo, ON N2L 3G1, Canada; 4 Department of Ophthalmology, University Hospital Leipzig, 04103 Leipzig, Germany; 5 Save Sight Institute, The University of Sydney, Sydney, NSW 2000, Australia

**Keywords:** drusen-like deposits, systemic lupus erythematosus, optical coherence tomography, choroidal vascularity index, optical coherence tomography angiography

## Abstract

Background: The relevance of drusen-like deposits (DLD) in patients with systemic lupus erythematosus (SLE) is to a large extent uncertain. Their genesis is proposed to be correlated to immune-complex and complement depositions in the framework of SLE. The intention of this study was to determine potential morphological differences in the choroid and retina as well as potential microvascular changes comparing two cohorts of SLE patients divergent in the presence or absence of DLD using multimodal imaging. Methods: Both eyes of 16 SLE patients with DLD were compared to an age- and sex-matched control-group consisting of 16 SLE patients without detectable DLD. Both cohorts were treated with hydroxychloroquine (HCQ) and did not differ in the treatment duration or dosage. Using spectral-domain optical coherence tomography (SD-OCT) choroidal volume measures, choroidal vascularity indices (CVI) and retinal layer segmentation was performed and compared. In addition, by the exploitation of optical coherence tomography angiography vascular density, perfusion density of superficial and deep retinal capillary plexuses and the choriocapillaris were analyzed. For the choroidal OCT-scans, a subset of 51 healthy individuals served as a reference-group. Results: CVI measures revealed a significant reduction in eyes with DLD compared to healthy controls (0.56 (0.54–0.59) versus 0.58 (0.57–0.59) (*p* = 0.018) and 0.56 (0.54–0.58) versus 0.58 (0.57–0.60) (*p* < 0.001)). The photoreceptor cell layer presented significant thinning in both eyes of subjects with DLD compared to control subjects without DLD (68.8 ± 7.7 µm vs. 77.1 ± 7.3 µm for right eyes, *p* = 0.008, and 66.5 ± 10.5 µm vs. 76.1 ± 6.3 µm for left eyes, *p* = 0.011). OCTA scans revealed no significant changes, yet there could be observed numerically lower values in the capillary plexuses of the retina in eyes with DLD than in eyes without DLD. Conclusions: Our results illustrated significant alterations in the choroidal and retinal analyzes, suggesting a correlation between DLD and the progression of inflammatory processes in the course of SLE leading to retinal degeneration. For this reason, DLD could serve as a biomarker for a more active state of disease.

## 1. Introduction

Systemic lupus erythematosus (SLE) is an autoimmune disease presenting itself in a heterogenous way with a multitude of clinical manifestations ranging from renal, cardiovascular, to ocular damage [1]. It is characterized by an elaborate set of immunologic processes, in particular the loss of immune tolerance and subsequent dysregulation by production of autoantibodies, formation of immunocomplexes, and activation of the complement system [1,2]. As the disease develops, systemic inflammation progresses leading to increasing organ destruction in a chronic relapsing course [2].

The etiology is yet unknown; the classification of SLE is based on criteria defined by the American College of Rheumatology (ACR) including various clinical and immunologic manifestations [1]. Although ocular involvement is not outlined in the diagnostic assessment [3], it can occur in up to 30% of SLE patients including conditions as keratoconjunctivitis, retinopathy, and optic neuritis among other ocular manifestations [1]. In the majority of cases, retinopathy and visual impairment manifests in already well-advanced disease states [4]. More rarely, choroidopathy has been detected [5], conceivably because of the limited technical possibilities to analyze the deeper more hidden part of the visual organ in the past.

Nowadays, more advanced optical coherence tomography (OCT) techniques enable us to perform in-depth evaluations of the retina as well as the choroid [6].

The involvement of the choriocapillaris has currently been subject of interest in SLE patients [6,7,8], as it appears to be influenced by disease activity [6]. The choroidal thickness seems to decrease in patients due to vasculitis and deposition of complement factors leading to a reduction in blood flow [6]. An upcoming method to examine the choroid, the choroidal vascularity index (CVI), is suggested to advance previous analyses of choroidal thickness by providing more stable results due to being less susceptible to physiological elements [9]. It could therefore be a potential new player in the evaluation of disease progression in SLE.

In recent years, the assessment of drusen-like deposits (DLD) has emerged without a considerable number of studies concerning this matter yet. Located between the Bruch membrane and the retinal pigment epithelium (RPE), there are differing hypotheses proposed regarding the potential value of DLD in disease process. Baglio et al. have found that DLD, detected by indocyanine green angiography (ICGA), only occur in SLE patients with lupus nephritis and having the anatomical commonalities of choroid and glomeruli in mind, stated that they could be an early marker for renal involvement [7]. Invernizzi et al., however, which made use of OCT, did not fully support that assumption, as they found DLD occurring in SLE patients without lupus nephritis as well [3]. They postulated it was more likely that the deposits are associated with complement pathway dysregulations taking place in the eye and in other capillary systems. On the other hand, the authors had discovered that the number, size, and distribution was significantly higher in SLE patients with renal involvement, correlating to the results found by Baglio et al. In conclusion, they claimed that DLD could be a marker for disease activity, as their presumed genesis accompanies the known pathophysiology of SLE and increases when renal involvement is present [3]. Former studies support this argumentation, as they found the presence of similar lesions as DLD to increase the threat of vision loss [10,11].

Ocular manifestations, as DLD and choroidal alterations, can be present without any noticeable symptoms and may therefore hold the opportunity to serve as a marker for further disease progression [4,10]

In this study, we aim to assess the morphological features in patients with SLE and drusen-like deposits using non-invasive imaging modalities and to compare them to age-and sex-matched group with SLE but without DLD and to a group of healthy subjects.

## 2. Methods

### 2.1. Study Design

Within the frame of a large-scale monitoring of patients treated with hydroxychloroquine (HCQ) at the Departments of Ophthalmology, University Hospital Zurich, from January 2012 to December 2020, patient data was extracted to be analyzed in a retrospective observational comparative cohort study. The trial is compliant with international norms as it was approved by the Institutional review board of Swiss ethics/BASEC (No. 2019-00972). A written informed consent was obtained from all patients participating.

### 2.2. Patient Inclusion and Exclusion Criteria

**Figure 1:**
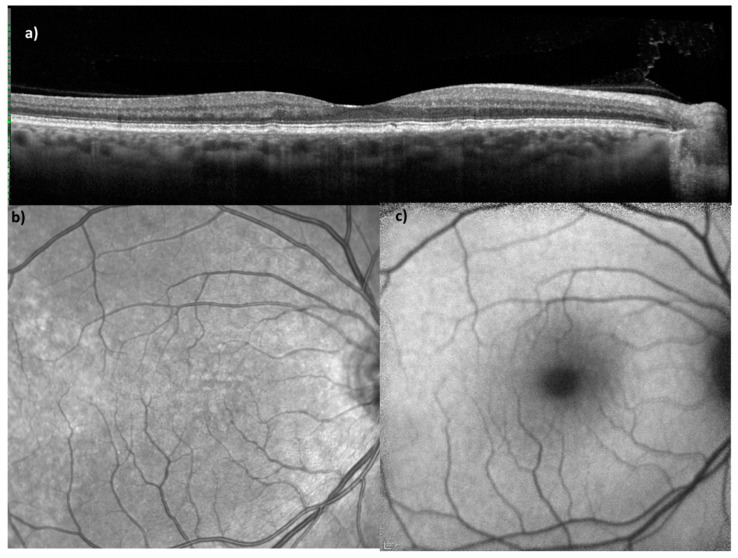
Multimodal imaging of a SLE patient with drusen-like deposits. (**a**) Illustrates an optical coherence tomography scan, (**b**) a near-infrared (NIR) image, and (**c**) a fundus autofluorescence image.

Subjects had to meet the following inclusion criteria: male or female patients with a confirmed diagnosis of systemic lupus erythematosus at an age ≥18 years were included from the HCQ clinic during regular screening exam. Furthermore, drusen-like deposits, defined as an accumulating precipitation leading to a detectable detachment of the RPE from the Bruch membrane, had to be verifiable in OCT (Figure 1). Patients were regularly seen in the course of routine HCQ-toxicity-screening including regular OCT-scans. A sufficient OCT and OCTA image quality had to be ensured to qualify for further analysis.

Evidence of other ocular or systemic diseases other than SLE, current or previous macular and retinal diseases, myopia > −3 diopters, and significant lens opacities were the exclusion criteria.

### 2.3. Data Collection

A cohort of 16 SLE-patients with documented drusen-like deposits were analyzed and compared to a control group including 16 SLE-patients without DLD, which were matched for age, sex, ethnicity, smoking status, dosage, and HCQ-treatment duration. HCQ-dosage data was collected by examination of the prescription on each consultation, which was cross checked by taking the patient’s medication history. Concerning the risk profile, an incidental significant difference in the prevalence of cardiovascular disease could be detected. Both the right and the left eye were scanned in the first exam and the last follow-up in order to identify potential alterations over time. The cohorts were further set against a healthy control group without retinopathy, systemic diseases involving the retina, or HCQ-intake. The probands were selected out of a pool of patients consulting our clinic due to various complaints and conditions, without influence to retinal structures, as for example conjunctivitis, complications concerning contact lenses, or cataract.

**Figure 2:**
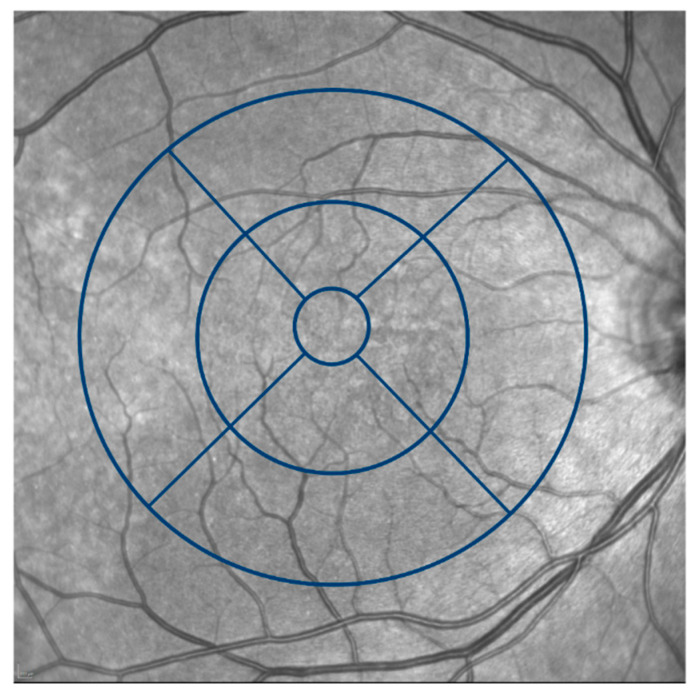
Grid for retinal layer thickness measurement. Retinal layer thickness was measured according to the ETDRS grid that was divided into a central area including the fovea with a diameter of 1mm, an inner ring with a diameter of 3mm, and an outer ring with 6 mm.

Spectral-domain optical coherence tomography (SD-OCT) enabled us to thoroughly analyze and assess differences in the retinal layers. An OCT scan and other imaging modalities of a SLE patient with DLD is illustratively demonstrated in Figure 1. The equipment was fully integrated with the analytical software, HEYEX 2, provided by Heidelberg Engineering (Heidelberg, Germany), which carried out an automatic segmentation. First, using ETDRS thickness maps, according to the ETDRS grid as shown in Figure 2, the software application automatically segmented the retinal layers into retinal nerve fiber layer (RNFL), ganglion cell layer (GCL), inner plexiform layer (IPL), inner nuclear layer (INL), outer plexiform layer (OPL), outer nuclear layer (ONL), and retinal pigment epithelium (RPE) (Figure 2). Thereafter, manual correction of potential artifacts risen from the automatic segmentation process was performed. Subsequently, the gathered data was compared to a healthy control group. For all layers analyzed, the thinnest and the thickest section in the centerfield of the ETDRS grid was determined, described as “Center Min” and “Center Max”, respectively.

**Figure 3:**
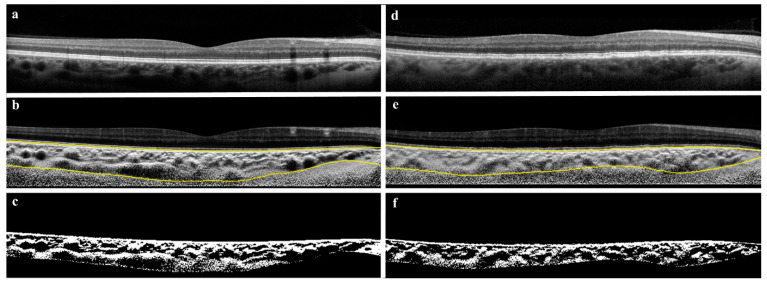
Choroidal vascularity index (CVI). CVI data was generated from OCT cross-sections (**a**,**d**) through automatic segmentation (**b**,**e**) and binarization (**c**,**f**). Pictured is a SLE patient without DLD (**left column**) and a SLE patient with DLD (**right column**).

Choroidal vascularity index (CVI) for the volume scans was calculated based on a previously reported algorithm. In brief, it involves shadow compensation, localization, and binarization of the choroid [12], as visualized in Figure 3. Shadow compensation reduces noise and increases the contrast enhancement of B-scans [13]. Choroidal segmentation was accomplished by identification of choroidal inner and outer boundaries shown as RPE-Bruch’s membrane and choroid-scleral interface, respectively. This was followed by binarization of choroidal vessels, wherein lumen of choroidal vessels was shown as dark areas and stromal areas were marked as bright areas. The ratio of choroidal luminal area to total choroidal area was defined as CVI.

Imaging by optical coherence tomography angiography (OCTA) was executed by PLEX Elite 9000 device, software version 2.0.1.47652 (Carl Zeiss Meditec Inc., Dublin, CA, USA). Centered on the fovea, images with the dimensions of 3 × 3 mm and 6 × 6 mm were obtained by a well-trained, certified ophthalmologist, whereas only the 3 × 3 mm-frames are presented in this study. Scans with a signal strength of 8 out of 10 or higher were included, as signal strength has been shown to influence quantitative measurements in OCTA [14]. Quantitative parameters of the 3 × 3-mm scans were automatically generated; layers of the superficial, deep retinal capillary plexuses and choriocapillaris were obtained using layer segmentation produced by the instrument software and prototype analysis vascular density quantification software (Macular Density v. 0.7.1, ARI Network Hub, Carl Zeiss Meditec Inc., Dublin, CA, USA) supplied by the manufacturer. The analyzed region of interest was the innermost ring of the ETDRS grid (inner ring, iR), with an inner diameter of 1 mm and an outer diameter of 3 mm, centered on the fovea. 

### 2.4. Statistical Analysis

Data was analyzed using Stata16 (StataCorp (2019) Stata Statistical Software: Release 16, College Station, TX, USA). Summary statistics included mean and standard deviation for continuous variables and frequencies with percentages for categorical variables. Clinical and OCT/OCTA data were reported as proportions, mean and standard deviation, or median and interquartile range for skewed data. Patients were divided into separate cohorts including SLE with DLD, SLE without DLD, and healthy controls. Data was compared between cohorts using chi-squared and Kruskal–Wallis tests. Statistical significance was defined as a *p*-value < 0.050.

## 3. Results

The demographics of the participants are illustrated in Table 1. There were no significant differences in the distribution of age, gender, ethnicity, or smoking status (*p* > 0.050). At the outset, the mean age in the study group was 41.2 ± SD (22–61) years, as compared to the control group without DLD 40.6 ± SD (20–61) years and the reference group of healthy people 57.7 ± SD (24–93) years. The majority of participants were female, as 12 out of 16 accounted for 66.7% of subjects in the study group and 11 out of 16, i.e., 68.8%, subjects in the control group were female. Regarding ethnicity, 13 patients of the DLD group were Caucasian and 3 were Asian; in the matched control group 10 were Caucasian, 5 were Asian, and 1 patient belonged to a different ethnic group. In the first group, five participants were smokers, four non-smokers, one person quit, and three had an unknown smoking status. The second group showed a similar tendency, with eight smokers, five non-smokers, one person who quit, and two individuals with an unknown smoking status.

Both SLE-patients with DLD and patients without DLD were treated with HCQ. As demonstrated in Table 2, no significant disparities in HCQ dose or treatment duration were found (*p* > 0.050). The median daily dose amounted to 200 mg in both groups with a mean cumulative dose of 567.6 g in the study group and 486.6 g in the control group. The dosage, measured in milligrams HCQ per kilograms bodyweight, was on average 3.8 mg/kg in probands with DLD and 4.3 mg/kg in probands without DLD. Treatment duration ran for 7.4 years and for 5.3 years in the case and the control group, respectively.

In regard to the comorbidities, there could be found a significant difference in the prevalence of cardiovascular diseases with 10 out of 16 subjects (62.5%) in the study group versus 3 out of 16 subjects (18.8%) in the control group. The distribution of renal involvement was homologous in both groups.

Table 3 compares choroid volume, bright and dark region volumes, as well as CVI. Information for eyes of SLE patients with DLD and SLE patients without DLD are demonstrated. The dataset was compared to a heathy control group. 

Patients with DLD revealed significantly lower values concerning the CVI than eyes of the healthy subjects, serving as a reference 0.56 (0.54–0.59) versus 0.58 (0.57–0.59) (*p* = 0.018) and 0.56 (0.54–0.58) versus 0.58 (0.57–0.60) (*p* < 0.001) for OD and OS, respectively. No significant difference in CVI could be observed comparing eyes of patients with DLD to eyes of patients without DLD. 

The choroid volumes, the bright region volumes, and the dark region volumes showed no significant alteration in comparison of the three demonstrated groups.

In addition to the analysis of the choroid, the retinal thickness of the different retinal layers was measured as shown in Table 4. 

The ganglion cell layer was significantly greater in eyes with DLD compared to eyes without DLD in the area with the highest measured thickness (36.9 ± 9.2 µm versus 28.6 ± 12.3 µm, *p* = 0.029).

The outer retinal layers and the photoreceptor cell layer demonstrated a significant thinning in eyes with DLD in contrast to eyes without DLD, opposing to the previously described tendency of thicker layers in the study subjects. The thinnest area of the ORL at last follow-up in the left eye showed a significant difference between the cohorts (81.0 ± 6.6 µm versus 84.5 ± 2.8 µm, *p* = 0.012). Concordantly, the layer of the photoreceptor cells presented significant thinning in both right and left eyes of study subjects compared to control subjects (68.8 ± 7.7 µm versus 77.1 ± 7.3 µm, *p* = 0.008 and 66.5 ± 10.5 µm versus 76.1 ± 6.3 µm, *p* = 0.011, respectively).

Table 5 shows the summarized data collected by optical coherence tomography angiography. The inner ring, in accordance to the ETDRS grid, was analyzed regarding the vessel density and perfusion density of the superficial and deep capillary plexus and regarding the choriocapillaris. 

While there were subtle numerical differences, with a tendency of decreased values in eyes with DLD when compared to eyes without DLD, there could not be found any significant changes.

## 4. Discussion

In this study, we investigated morphological alterations in the retina and choroid as well as changes in vasculature depending on the presence of drusen-like deposits in patients with systemic lupus erythematosus. For this purpose, the choroidal volumes, choroidal vascularity indices, retinal layers, and optical coherence tomography angiography images of SLE patients with documented DLD were compared to sex- and age-matched controls and a subset of healthy individuals.

Eyes of SLE patients showed an overall reduction in choroid volumes compared to healthy subjects and a noticeable tendency could be observed with greater volumes held by eyes with DLD than eyes without DLD. Furthermore, DLD eyes revealed a significant decrease in CVI. The retinal layer analysis demonstrated a trend leaning to a thickness increase of the retinal segments in the study subjects. Conversely, the photoreceptor cell layer illustrated a significant thinning in patients with DLD. Evaluating the OCTA analysis, there was an overall leaning to lower values in the capillary plexuses of the retina in eyes with DLD.

In our investigations, the choroid volumes tended to be greater in patients with DLD than in patients without; still, when set against healthy subjects, a general decrease was observed. Altinkaynak et al., who examined the choroidal thickness in SLE patients, detected a thinner choroidal layer and stated that this could be a consequence of vasculitis and immune-deposits leading to a decrease in blood flow with following atrophy in the choroid [6]. This could be an explanation for the tendency of lower choroid volumes in diseased patients in our cohort but does not explain why eyes with DLD show higher values than eyes without DLD. More clarification in this regard can be brought by Invernizzi et al., who found thicker CTs in patients with SLE compared to controls and proposed that this finding was due to the known relationship between choroidal thickness and systemic inflammatory conditions in SLE. They suggest that DLD could be a factor for active disease and that an active inflammatory state is the cause for the thicker CTs [3]. While atrophy through capillary luminal obstruction leads to a thinning, as shown by Altinkaynak et al., where an inclusion criterion was an inactive state of SLE, active inflammation leads to thicker choroids, i.e., through inflammatory cell infiltration [3,6,7].

In the present study, CVI was significantly lower in patients with DLD in comparison to healthy controls. On the first glance, this seems not to be concomitant with the detected higher choroid volumes. However, if considering that CVI as opposed to the absolute volume measures is a relative value resulting from the division of the luminal area to the total choroidal area, consisting of the luminal and the stromal area, there is a possible explanation. SLE is an inflammatory disease affecting primarily the capillary systems and the connective tissue [3,15]. As inflammation progresses with activation of inflammatory cytokines, antibody, and cell infiltration, the stromal area expands by accumulation, simultaneously a vascular occlusion by deposition of immunocomplexes and complement factors lead to a decreasing diameter of the vascular lumen of the choroid [6,16]. In conclusion, the CVI can decrease without a decline of the absolute choroidal volume values. To our knowledge, there is only one other study examining the CVI in SLE patients with the juvenile type, who found no significant difference between diseased and healthy probands. They justified their results by concurrent inflammatory deposition in the stroma and vasodilation [15], though it can be assumed that patients with juvenile SLE do not show much degenerative changes in the vasculature yet.

Consistent with the results from Dias-Santos et al. [17], we observed that the photoreceptor cell layer was significantly thinner in SLE patients with DLD compared to patients without DLD. As a tissue with high metabolic needs, the function of the cones and rods is directly dependent on the neighboring nourishing choriocapillaris [6,17,18]. As described above, SLE can involve the choroidal structures and blood flow and thus, leading to ischemia and atrophy in the retinal segments they supply with oxygen and nutrients [17]. In this context, our results could suggest that DLD, as a possible expression of choroidopathy, could be a sign of advancing disease [8].

Our results show that retinal layers and the retina itself are thicker in SLE patients with DLD compared to patients without. This comparison is the first to our knowledge. When assuming that DLD are a sign of advancing disease, this finding stands in contrast with the known literature. Previous studies mostly state a thinning of the retinal layers in patients diagnosed with SLE compared to healthy controls nota bene [17,19,20]. Liu et al. reason, that this thinning is due to vasculitis with subsequent atrophy in the ganglion cell layer [20]. Dias-Santos et al. suggest there is retinal neurodegeneration taking place in the course of SLE disease progression. However, the authors observed that the layer increases in thickness as SLE advances, justifying this with neuronal remodeling [17]. Investigated and discussed in more detail by Jones et al., who examined the development of neurodegeneration in the retina, illustrated that after initial retinal damage, as for example in retinis pigmentosa or AMD, cell death with loss of neurons, reactive unstructured neuritogenesis, reformation of new synaptic connections, and glial reaction come to pass [21]. This remodeling is seen as a part of the degenerative process and could be affecting most of the retinal layers [21]. Furthermore, in a histopathological examination of a SLE patient, an infiltration of macrophages into the retina could be proven [22]. Those inflammatory and degenerative processes in SLE could, to a certain extent, be a possible explanation on why thicker retinal layers could be measured in the current study.

Drusen-like deposits seem to differ from AMD-derived drusen regarding the pathogenesis. Drusen develop by a progredient accumulation of lipoproteins, especially in the macular Bruch membrane [23]. As further proteins and lipids precipitate, conductivity of the membrane becomes poorer leading to oxidative stress and subsequent inflammation mostly manifesting with advanced age. This process is additionally promoted by different genetical and environmental parameters [23,24].

The mechanism of formation of DLD is not fully clear; it is assumed that depositions of immunocomplexes and alteration of the complement system are playing a major role [3,6]. Further points to reinforce this assumption is the age of manifestation, as DLD appear to occur in diseases affecting younger patients, seen in SLE in the present study [1] and other diseases, where the complement system is involved [25,26]. In addition, the distribution differs from drusen; while drusen do not spare the fovea, DLD are observed to mainly present in a perifoveal arrangement leaving the centerfield out [3]. Once more, this is supported by observations in diseases other than SLE, where immunocomplex and complement activation are implicated [25]. The fact that drusen-like deposits and similar lesions are detected in SLE as well as in primarily renal diseases strongly suggest a shared susceptibility to similar pathophysiologic triggers due to the anatomical and functional resemblance of the glomerular system and the Bruch membrane-RPE complex [7]. In addition, in patients with lupus nephritis, the manifestation of DLD was significantly more pronounced [3], further indicating a prognosticating value of these ocular lesions for disease severity and progression. Another aspect to be mentioned is that so-called silent lupus nephritis was frequently detected by renal biopsies but did not show any alteration in conventional urinalysis or laboratory findings [27], thus assigning OCT a leading role as a possible screening method to identify patients at risk for renal and visual impairment.

In regard to the OCTA analysis, the present study showed no significant results, yet an apparent reduction in the VD and PD of both superficial and deep retinal capillary plexuses of patients with DLD. This finding goes in line with the earlier literature, as the studies we investigated all showed a decrease in retinal microvascular density in SLE patients [19,28,29,30,31,32]. An altered capillary blood flow causes microangiopathy and subsequently, if left untreated, vasoocclusion with retinal damage and threat to visual acuity [28,31]. Microangiopathy is the most common etiology of lupus retinopathy, which in turn is the most common cause for visual impairment in SLE patients and is furthermore associated with poorer survival prognosis [4,29]. VD measurement is suggested to serve as a potential biomarker for SLE disease activity, as they exhibit a significant negative correlation to each other [29,30]. A possible explanation for the non-significant results in the current study could be provided by the suggested protective effect of HCQ concerning the VD, as Conigliaro et al. found a positive correlation between superficial and deep retinal vessel densities and HCQ intake [32]. All of our enrolled SLE patients were administered to lower dosages of HCQ than in the study from Conigliaro et al. [32].

Limitations to our study include the limited number of probands enrolled. Furthermore, retinal layers and OCTA images had no control group consisting of healthy individuals, who could serve as reference. No involvement or correlation of disease-activity scores such as the systemic lupus erythematosus disease activity index (SLEDAI), which could possibly deliver more validity to our statements, were included. In addition, a correlation analysis between DLD and systemic alterations, according to the individual classification criteria of the ACR, was not pursued; however, this would be an interesting point to investigate in more detail in future research.

In conclusion, SLE patients with DLD seem to be in a more active state of disease as compared to patients without such lesions. Supported by our study results, the choroid volumes tend to be greater and the CVI to be lesser in patients with present DLD as a likely consequence of an active inflammatory state including vasculitis, immunocomplex, and complement depositions in the capillary systems, all characteristics in the pathophysiology of SLE. Subsequently, as our findings illustrate degenerative processes in the retina follow with a thinning of the photoreceptor cell layer and a decrease in retinal blood flow.

## Figures and Tables

**Table 1:** Baseline demographics. SD, standard deviation.

	Eyes with DLD (*n* = 16)	Eyes without DLD (*n* = 16)	Healthy Control Group (*n* = 51)
Age, years, mean (SD)			
First Exam	41.2 (12.3)	40.6 (12.3)	57.7 (18.3)
Sex, *n* (%)			
Male	4 (33.3)	5 (31.3)	24 (47.1)
Female	12 (66.7)	11 (68.8)	27 (52.9)
Ethnicity, *n* (%)			
Caucasian	13 (81.3)	10 (62.5)	
Asian	3 (18.8)	5 (31.3)	
Other	0 (0.0)	1 (6.3)	
Smoking Status, *n* (%)			
Yes	5 (38.5)	8 (50.0)	
No	4 (30.8)	5 (31.3)	
Quit	1 (7.7)	1 (6.3)	
Unknown	3 (23.1)	2 (12.6)	

**Table 2:** HCQ treatment characteristics and prevalence of relevant comorbidities. Significant differences shown in bold type. SD, standard deviation.

	Eyes with DLD (*n* = 16)	Eyes without DLD (*n* = 16)	*p*-Value
HCQ Treatment			
Daily Dose, mg (range)	200 (200–200)	200 (200–400)	0.260
Cumulative Dose, g (range)	567.6 (450.5–899.8)	486.6 (322.4–1026.5)	0.429
Dosage, mg/kg (range)	3.8 (2.8–5.1)	4.3 (2.7–5.3)	0.612
Treatment Duration, years (range)	7.4 (4.7–12.3)	5.3 (2.5–8.3)	0.188
Comorbidity, *n* (%)			
Cardiovascular Disease	10 (62.5)	3 (18.8)	**0.036**
Renal Involvement	3 (18.8)	2 (12.5)	0.678
GFR 1, mean (SD)	88.1 (33.7)	101.4 (18.0)	0.321
GFR 2, mean (SD)	90.6 (32.7)	96.4 (31.4)	0.856

**Table 3:** Choroid volume analysis and choroidal vascularity indices. Significant differences shown in bold type.

		Eyes with DLD (*n* = 16)	Eyes without DLD (*n* = 16)	Healthy Control Group (*n* = 51)
Bright Region Volume WSR, mm^3^, mean (range)	OD	0.62 (0.54–0.76)	0.49 (0.35–1.06)	0.77 (0.48–0.90)
	OS	0.57 (0.46–0.72)	0.49 (0.39–1.18)	0.57 (0.49–0.81)
Dark Region Volume, mm^3^, mean (range)	OD	0.26 (0.24–0.32)	0.22 (0.15–0.42)	0.31 (0.21–0.36)
	OS	0.25 (0.21–0.31)	0.20 (0.16–0.50)	0.24 (0.19–0.32)
CVI WSR Proposed, mean (range)	OD	0.35 (0.28–0.43)	0.26 (0.20–0.63)	0.44 (0.29–0.52)
	OS	0.32 (0.25–0.40)	0.28 (0.21–0.67)	0.34 (0.26–0.48)
Bright Region Volume WSR, mm^3^, mean (range)	OD	**0.56** **(0.54–0.59)**	0.57 (0.52–0.58)	**0.58** **(0.57–0.59)**
	OS	**0.56** **(0.54–0.58)**	0.56 (0.55–0.58)	**0.58** **(0.57–0.60)**

OD oculus dexter, OS oculus sinister, CVI choroidal vascularity index, WSR with shadow removal.

**Table 4:** Retinal layer analysis. Significant differences shown in bold type. SD, standard deviation.

		Eyes with DLD		Eyes without DLD	
		OD	OS	OD	OS
Retina, µm	Center Min ± SD	222.6 ± 20.2	224.3 ± 14.0	205.1 ± 59.4	217.3 ± 25.4
	Center Max ± SD	318.4 ± 24.9	321.8 ± 17.7	291.2 ± 82.5	313.8 ± 27.1
Ganglion Cell Layer, µm	Center Min ± SD	1.4 ± 1.5	0.8 ± 1.5	1.1 ± 1.2	1.3 ± 1.2
	Center Max ± SD	**36.9 ± 9.2**	37.9 ± 11.0	**28.6 ± 12.3**	32.5 ± 10.5
Outer Nuclear Layer, µm	Center Min ± SD	55.3 ± 14.9	50.7 ± 13.9	51.6 ± 19.3	54.5 ± 17.1
	Center Max ± SD	117.8 ± 12.4	117.1 ± 14.7	102.4 ± 32.3	108.5 ± 17.1
Retinal Pigment Epithelium, µm	Center Min ± SD	11.4 ± 1.9	11.4 ± 2.6	11.6 ± 3.5	12.5 ± 2.2
	Center Max ± SD	28.1 ± 6.9	36.1 ± 23.1	23.6 ± 6.7	25.6 ± 4.2
Outer Retinal Layers, µm	Center Min ± SD	81.0 ± 5.5	81.5 ± 5.3	79.8 ± 21.9	84.4 ± 5.7
	Center Max ± SD	98.4 ± 7.8	104.6 ± 18.4	95.9 ± 26.5	101.7 ± 8.6
Photoreceptor Cell Layer, µm	Center Min ± SD	69.7 ± 5.8	69.5 ± 4.5	72.7 ± 5.5	71.9 ± 5.3
	Center Max ± SD	**68.8 ± 7.7**	**66.5 ± 10.5**	**77.1 ± 7.3**	**76.1 ± 6.3**

OD oculus dexter, OS oculus sinister.

**Table 5:** Vascular analysis using OCTA. SD, standard deviation.

		Eyes with DLD	Eyes without DLD	*p*-Value
3 × 3 Mean Inner Ring, mm/mm^2^, mean (SD)	Vessel Density Superficial	16.4 (2.4)	17.6 (1.1)	0.248
	Vessel Density Deep	12.7 (3.3)	13.1 (1.6)	0.847
	Perfusion Density Superficial	0.37 (0.05)	0.39 (0.02)	0.501
	Perfusion Density Deep	0.27 (0.07)	0.28 (0.04)	0.773
	Choriocapillaris Area	1.6 (0.0)	1.6 (0.0)	1.000
	Choriocapillaris % Area	18.1 (5.1)	18.1 (4.3)	0.700

## Data Availability

Full access to the data is possible by the corresponding author.
